# A Great Match and a Memorable Day

**Published:** 1920-03-20

**Authors:** 


### THE HOSPITALS' RUGBY FINAL.

### A Great Match and a Memorable Day.

One of the first signs of peace and its effect on the lighter side of hospital life was seen on Thursday, March 11, when Guy's, by beating St. Bartholomew's by two tries (6 points) to nil in the final at Richmond Athletic Ground, won the Inter-Hospitals Rugby Football Cup. As one reported remarked, so many things happen before and during—and after!—a Hospitals Cup final, that it is a little difficult to know where to begin a description of what must have been a truly eventful day for those participating, directly or indirectly, in the conflict. Several events were, however, outstanding. The presence of the King among a crowd which constituted a record in both numbers and enthusiasm lent an atmosphere to the game itself which is rarely, if ever, seen outside this country. The demonstrations and touch-line antics of literally hundreds of students in all manner of fancy costumes provided a scene which can be likened to nothing else in this world. A procession extending completely round the ground included, besides most of the youth of the hospitals actually concerned, not only girl students from the Royal Free Hospital with banner and jester complete, but sections from many other London medical units. True, the costumes were not always eccentric, but in numbers, a more remarkable and happy demonstration of the strength of the youthful medical element in London could scarcely be imagined. To describe these proceedings as a "rag," or to view them in the same light as the sundry acts of semi-hooliganism for which a few isolated and singularly ill-advised batches of students, non-medical we believe, have recently been responsible is a gross injustice, and if suppression of this harmless touch-line buffoonery is ever attempted, we should deplore the loss of one of the most picturesque football events of the season. Nothing better could be asked than the respect granted to Royalty and the consideration shown for the interests of spectators and players alike. The sight of the teams lined up for inspection by His Majesty and backed by row upon row of blazingly coloured, cheering fellow-students is one to be remembered for many a day.

Of the game itself excellent reports have already appeared, and, as is usual in these conflicts, simply a remarkable scramble was witnessed, in which skill was largely overshadowed by vigour and sheer individual determination. Guy's Hospital have an undoubtedly clever team, with an almost great reputation, but in "cup-tie" football they nearly found their match in St. Bart's. In fact, had the latter taken half their chances the result might easily have been reversed. Nevertheless, as play went the better side won, and certainly the Guy's backs when they did for a moment get going proved their superiority. The first try came early as the result of an excellent open movement, P. Krige scoring far out on the left, and about this period and later in the second half, after the Guy's forwards had forced the second score, the winners showed the only glimpses of that power to play really clever football which they undoubtedly possess. St. Bartholomew's are to be congratulated upon one of the finest exhibitions of hustling and spoiling one could ever wish to see. Just a little more luck and a little more judgment and reward might have been theirs; but after all, neither set of men were new to hospital football or new to the hospital cup-tie spirit. May it long survive! At the close of the game the ground once more echoed to cheer upon cheer as the teams were first carried shoulder-high to the pavilion, and again when His Majesty presented the famous cup to W. D. Doherty, the Guy's captain. An innovation was provided by the presence of a biplane, which hovered low over the ground. Was it coincidence that its colour also was orange and blue, in accordance with the prevailing tints of the extravaganza below?

And so the day had started well. Winners and losers could both congratulate themselves, the returning processions through London eclipsed in every way any similar pre-war festivities, and for the evening, in honour of the youths' return to hospital student life, the whole house of the London Hippodrome had been booked up by the two institutions concerned. The stalls were packed with the revellers of the afternoon, now shorn of their grease-paint and colours and providing a pulsating study in black and that glittering white which only the conventional shirt-front can convey. An excellent revue performance was at times helped rather than interrupted by the vocal attentions of an enthusiastic audience, and the sight of Mr. George Robey "dealing," if one may so use the term, with the combined wits of several hundred of England's future doctors was a sight worth going miles to see—and indeed stamps him as an artist of the final and first degrees.

Of rowdyism there was practically nil. The hospitals' medical staff, with their wives and friends, attended the theatre in force. True, they occupied the dress circle as a precautionary measure, but none could take exception to the events of an evening where harmless fun was the only fashion. A packed house enjoyed an absolutely unique experience, and with the final fall of the curtain came to an end a day on which the medical student and the youth of the hospital world gave London once more to know that an ounce of mirth is worth a pound of sorrow and yet left everywhere a trail of unblemished good-will behind them.

